# Public-Private Sector Mix Approach to Achieving Effective, Efficient and Value-Added TB Programming in Nigeria: Lessons Learned

**DOI:** 10.3389/ijph.2024.1606807

**Published:** 2024-02-13

**Authors:** Ifeanyi Okekearu, Onyinye Ojeh, Kenneth Okoineme, Jane Adizue, Yusuf H. Wada, Elizabeth Adeyemo, Jennifer Anyanti, Abdullahi Musa Yola, Abubakar Sadiq Umar

**Affiliations:** Society for Family Health Nigeria, Abuja, Nigeria

**Keywords:** public-private mix, tuberculosis, PPM, TB, Nigeria

## Background

Among all infectious diseases, tuberculosis (TB) is the leading cause of death worldwide, and Nigeria ranks second in Africa and sixth out of the eight nations with the highest TB burden globally [1]. In Nigeria, TB treatment is mostly through public health facilities [2], even though the private health sector remains the first point of call for over 60% of Nigerians [3]. Private health facilities include clinics, standalone medical laboratories, community pharmacies, nursing homes, proprietary patent medicine vendors (PPMV), faith-based clinics, traditional/herbal homes, and others. In a public-private sector-driven health system such as Nigeria, private health providers are very important stakeholders in tuberculosis diagnosis and management. The level of collaboration and involvement of the private health sectors in Nigeria in TB case findings, treatment, and notification remains below expectations, little wonder why Nigeria is rated as one of the top 4 countries in the world with missing TB cases [4]. Therefore, Society for Family Health, through its TB LON 1&2 project provides recommendations in advancing actions towards an active private-public mix (PPM) approach to achieving effective and efficient TB management in Nigeria.

## Nigerian TB Context and the Private Sector

Nigeria was among the very few countries that recorded an increase in TB notifications in 2020 at the height of the COVID-19 pandemic, the attendant lockdown and other measures introduced to combat the pandemic [5]. These affected all spheres of human endeavour, including health services. The country, since 2019, has recorded a significant and consistent increase in the annual TB notification [4]. Nigeria recorded a massive increase in the annual TB notification in 2021 when the TB case notification increased by 50% from 138,591 in 2020 to 207,785 TB cases in 2021. Many factors are responsible for this progress, including the rising consciousness and efforts by private healthcare providers and facilities in case finding, treatment and notification [4]. The World Health Organisation (WHO) has emphasized the significance of the public sector playing a stewardship role in involving private care providers in efforts to achieve the public health goal of tuberculosis control [1]. Considerable evidence has shown that PPM is successful in reducing patient burden, increasing case notification, and improving treatment outcomes [5]. For example, a cross-sectional study highlighted that, in comparison to public health facilities in Kaduna State, private facilities comply with national guidelines, have a higher case load of TB patients, and record better treatment outcomes [6]. While patient contact screening was extremely low in both facilities, records were better completed in private facilities [7]. In a similar study conducted in India, PPM was reported to have resulted in a 12% increase in TB case notifications [8]. TB case notification and treatment success rates were significantly enhanced in Myanmar when private general practitioners (GPs) were involved in tuberculosis management [9]. Overall, these studies highlight the need for countries to utilize the full capacity of all healthcare providers in either public or private facilities [10].

## Recommendations

Based on the identified challenges and recognising the importance of the PPM in strengthening TB programming, we provided recommendations as priority actions in line with the established Task Shifting and Task Sharing (TSTS) policy in Nigeria [7].

### Promote and Support the Private Health Sector and Providers to Participate More in Provider-Initiated Mobilisation and Case Findings

Screening allows detecting tuberculosis in the latent stages of its development and undertaking timely measures to prevent it from transitioning into the active and contagious stage. In this way, it will be possible to minimise the costs associated with the treatment of the infection. To eradicate tuberculosis in Nigeria, efforts by the government agencies such as NTBLCP, in collaboration with other relevant actors, such as donors and the private sector, must be directed at the prevention of the disease through awareness-raising activities and promotion of screening exercises.

### Institutional Strengthening and Capacity Support for Private Providers and Facilities to Have Requisite Knowledge and Capacity for TB Programming

The knowledge of risk factors contributing to the development of tuberculosis, as well as positive individual and public attitudes to this infection, are linked to engagement in protective behaviours and favourable perceptions of such preventive practices as screening. In contrast, the lack of knowledge and tuberculosis-related stigma characterized by perceived incurability, beliefs about the etymology of the infection, and negative views on associations of tuberculosis with HIV often result in non-disclosure of the disease, non-compliance with treatment, low self-esteem, ridicule, social exclusion, and so forth. The government’s role through the NTBLCP efforts should also be channelled towards strengthening institutions and providing capacity support for private providers to improve TB programming.

### Strengthen Policies to Promote Effective PPM

While the government has acknowledged that to promote greater private sector involvement in the provision of public services and draw private capital to finance the nation’s infrastructure and related services, it will need to strengthen its policies and practices to advance the cause. Actions such as offering a clear institutional and policy framework for PPM and effectively communicating its policies to the policymakers, the public, and investors will go a long way in creating an enabling policy environment for PPM.

### Resourcing

While commitment from donors to support efforts to increase local ownership and sustainability of the TB response in Nigeria exists, efforts to build the capacity of private providers at a local level and help communities tailor their own solutions to TB prevention and treatment need to be intensified. Substantial efforts to enhance the procurement and supply chain management system, health management information systems, surveillance systems, human resources for health, governance and financing, capacity building and incentive for the private sector are imperative. The incorporation of TB management in the range of treatments offered by the National and State Health Insurance Schemes, as well as the 1% consolidated revenue of the Basic Healthcare Provision Fund (BHCPF), should be leveraged. The government should push for a formal involvement of the State Oversight Committees across states to acquaint them with the BHCPF implementation and encourage them to fulfil its intended objectives.

### Incentivising PPM

Effective PPM for tuberculosis management has been found to thrive on the availability of several factors, including incentives [4]. Patient and provider involvement in TB case detection, diagnosis, and treatment can be encouraged by utilising incentives to engage private providers. To create verifiable and trustworthy incentive systems, donors will need useful insights about providers’ and users’ microeconomics and motivations. For providers, maintaining an active TB practice is not financially rewarding and may even be self-defeating. Thus, financial incentives can encourage them to increase their understanding or practice of managing TB. Other non-financial incentives can be utilised such as awarding, certifying, or recognizing service providers. This kind of distinction could help private service providers stand out from competitors and broaden their clientele. Using moral persuasion to influence a provider’s sense of social duty and providing access to discounted health supplies, networking opportunities, and capacity building sessions are also proven incentives for promoting effective PPM.

## Conclusion

While Nigeria is making progress with TB case finding and notification, it is imperative to scale the PPM to enhance the chances of finding thousands of missing TB cases. We have provided recommendations which serve as key instruments for a more cohesive and integrated health system.
